# Renal Artery Dissection in an Emergency Department Patient With a Near Fall

**DOI:** 10.7759/cureus.36759

**Published:** 2023-03-27

**Authors:** Susannah Boulet, Melody L Milliron

**Affiliations:** 1 Emergency Medicine, Allegheny Health Network-St Vincent Hospital, Erie, USA

**Keywords:** vascular injury, nephrectomy, near fall, renal artery, dissection

## Abstract

51-year-old male presented to the emergency department with left flank pain after a near fall on steps. Computed tomography of the abdomen and pelvis with contrast showed a non-enhancing left kidney, secondary to suspected acute traumatic dissection of the left renal artery.

Renal artery dissection is typically affiliated with blunt abdominal trauma, though it can also occur spontaneously. The diagnosis of a renal artery dissection after minor trauma can often go unrecognized due to a lack of initial severe symptoms.Management will vary upon the age of the injury, the preservation of the kidney, and the extent of associated injuries.Ultimately, management should be dictated by discussion with trauma surgery, vascular surgery, urology, or interventional radiology consultants. Knowing the mechanism of injury and patient risk factors can help guide your ability to successfully identify and treat the patient, limiting delays in care and potentially lowering the incidence of organ injury.

## Introduction

Renal artery dissection is typically affiliated with blunt abdominal trauma but can be spontaneous, most commonly due to a connective tissue disorder [1]. The diagnosis of a renal artery dissection after only a minor trauma can often go unrecognized due to a lack of initial severe symptoms [2]. Management varies depending on the age of the injury, kidney preservation, and the extent of associated injuries [2,3].

Renal artery dissection is a diagnosis that can be made and initially managed in the emergency department. Knowing the mechanism of injury and patient risk factors can help guide your ability to successfully identify and treat the patient, thereby limiting delay in care and, ultimately, the potential of minimizing the risk of renal injury.

## Case presentation

**Figure 1:**
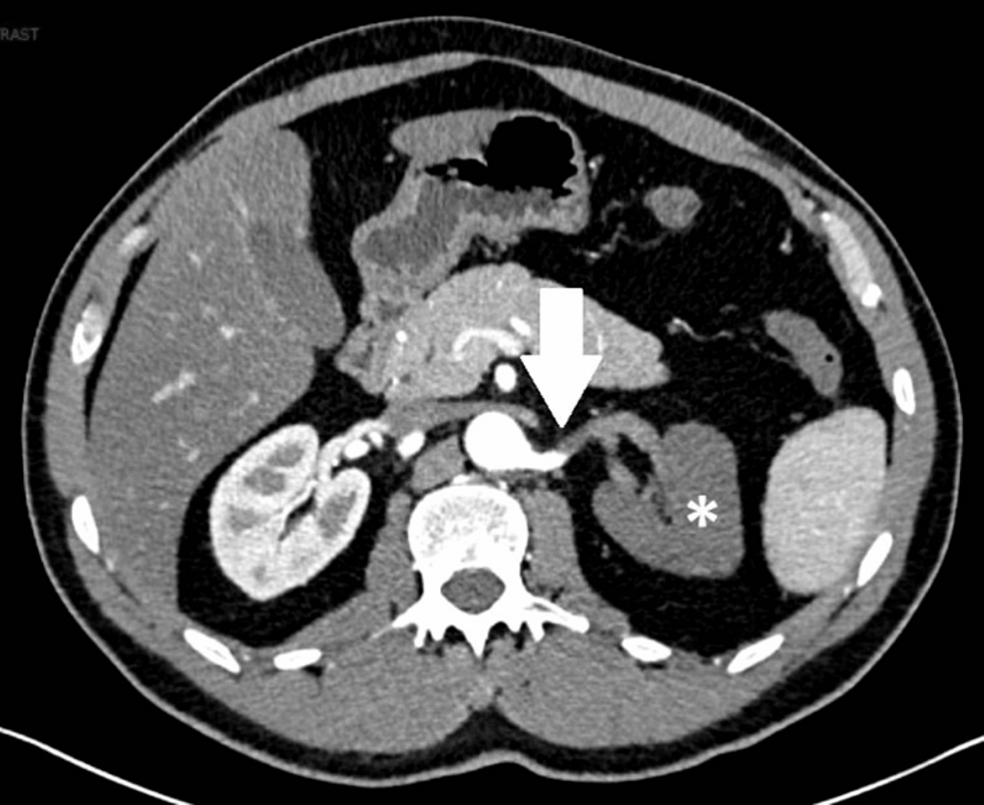
Computed tomography of the abdomen and pelvis with intravenous contrast in a 51-year-old male with left-sided abdominal pain. Arrow identifies non-enhancement of the left middle to distal renal artery, secondary to traumatic dissection. Asterix identifies the left kidney as lacking enhancement.

**Figure 2:**
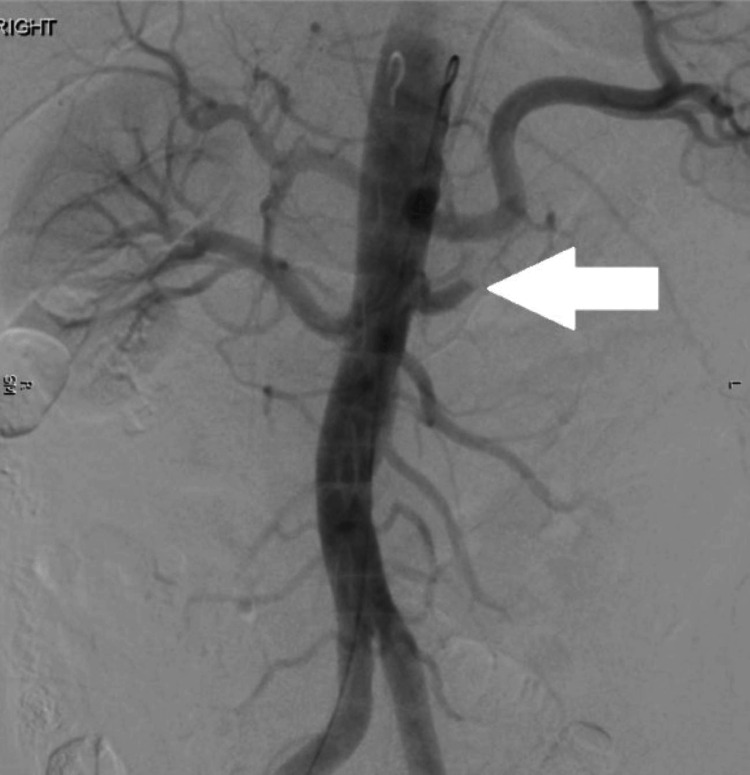
An angiogram confirms the lack of flow to the left renal artery, which is consistent with renal artery dissection. The arrow identifies the site of suspected renal artery dissection.

A 51-year-old male presented to the emergency department with abdominal pain after walking downstairs, slipping on clothing, and twisting backward without falling or striking the ground. He was initially pain-free but later developed left-sided flank pain with associated nausea and vomiting at work and was unable to sit without discomfort. He denied taking any anticoagulation medications or additional other traumatic events. On physical examination, the patient was observed to be pacing in the examination room. Abdominal examination revealed significant left upper quadrant tenderness with an otherwise soft abdomen. Due to concern for intrabdominal injury, computed tomography (CT) of the abdomen and pelvis with intravenous contrast was obtained. CT revealed left renal non-enhancement, secondary to suspected acute traumatic left renal artery dissection (Figure 1). A discussion with the trauma surgeon and vascular surgeon at an outside facility occurred. Due to the lack of other injuries, the decision was made to initiate heparin anticoagulation in an attempt at renal salvage. The patient was emergently transferred for a higher level of care, and an angiogram (Figure 2) showed a complete lack of flow to the left kidney. Unfortunately, the patient ultimately required a nephrectomy due to renal necrosis.

## Discussion

The most common complaint after a renal injury is ipsilateral flank pain [4]. The clinical presentation with acute flank pain and hematuria may be initially misleading to the provider and guide them towards a CT without intravenous contrast to evaluate for ureteral calculi [5]. This could lead to a delay in obtaining a time-sensitive diagnosis for renal salvage.

Renal injuries, including vascular injuries, contusions, or lacerations, occur in 1% to 5% of blunt abdominal trauma patients [4]. Renal arterial injury is rare. Renal artery dissection is associated with acceleration and deceleration injuries [3]. Dissection results from rapid body motion forces combined with the kidney’s anatomic mobility. This results in a stretch lesion and an intimal tear [2]. Although these are rare injuries, the vascular injury should be included in the differential diagnosis of flank or abdominal pain after rotational or twisting forces without subsequent direct trauma. CT with contrast or angiography of the abdomen and pelvis is the initial imaging modality of choice.

Injury to the renal artery following blunt trauma is now readily detected due to the use of CT with intravenous contrast, but the optimal treatment is controversial due to a lack of current guidelines [2]. Five main traumatic renal artery dissection patterns have been described: avulsion of the renal hilum, dissection of the segmental renal branches, preocclusive main renal artery dissection, renal artery stenosis without flow limitation, and thrombogenic renal artery intimal tears [2].

In the trauma patient, management depends on a variety of factors, including the patient’s overall injuries and renal injury time at diagnosis. Non-operative management is preferred in non-flow-limiting renal artery dissection, and catheter-directed evaluation and embolization are important additions in cases of active bleeding [2]. The therapeutic window for kidney revascularization with complete loss of renal flow may be variable. Endovascular stenting beyond four hours after trauma is typically only recommended if intact renal parenchymal perfusion is detected at angiography. Antiplatelet therapy may be recommended after cases of stenting, but only after the calculation of the patient's bleeding risk [2].

The treatment of renal artery dissection lacks consensus guidelines, and interventional options may include balloon angioplasty or stenting [1,2]. Conservative medical management may include anticoagulation, antiplatelet agents, antihypertensives, and pain control [1]. Management should be guided by discussions with urology, vascular surgery, and interventional radiology.

## Conclusions

While solid organ injury should be highly suspected in a patient presenting to the emergency department with trauma, our rare case reminds us that it is important to keep vascular injuries on the differential diagnosis list. The diagnosis of a renal artery dissection after minor trauma can often go unrecognized. Unfortunately, in this case, the delayed presentation decreased the likelihood of renal salvageability. Management will vary on the age of the injury, the preservation of the kidney, and the extent of associated injuries. Knowing the mechanism of injury and patient risk factors can help guide your ability to identify the patient's injuries and minimize the risk of renal injury.
